# Publishing perishing? Towards tomorrow's information architecture

**DOI:** 10.1186/1471-2105-8-17

**Published:** 2007-01-19

**Authors:** Michael R Seringhaus, Mark B Gerstein

**Affiliations:** 1 Department of Molecular Biophysics and Biochemistry, Yale University, 266 Whitney Avenue, New Haven, CT 06520, USA; 2 Program in Computational Biology and Bioinformatics, and Department of Computer Science, Yale University, 266 Whitney Avenue, New Haven, CT 06520, USA

## Abstract

Scientific articles are tailored to present information in human-readable aliquots. Although the Internet has revolutionized the way our society thinks about information, the traditional text-based framework of the scientific article remains largely unchanged. This format imposes sharp constraints upon the type and quantity of biological information published today. Academic journals alone cannot capture the findings of modern genome-scale inquiry.

Like many other disciplines, molecular biology is a science of facts: information inherently suited to database storage. In the past decade, a proliferation of public and private databases has emerged to house genome sequence, protein structure information, functional genomics data and more; these digital repositories are now a vital component of scientific communication. The next challenge is to integrate this vast and ever-growing body of information with academic journals and other media. To truly integrate scientific information we must modernize academic publishing to exploit the power of the Internet. This means more than online access to articles, hyperlinked references and web-based supplemental data; it means making articles fully computer-readable with intelligent markup and Structured Digital Abstracts.

Here, we examine the changing roles of scholarly journals and databases. We present our vision of the optimal information architecture for the biosciences, and close with tangible steps to improve our handling of scientific information today while paving the way for an expansive central index in the future.

## The Goldilocks Effect: Why Journals Drop Big (and Small) Results

The scale of biological data varies tremendously and encompasses a wide array of results: from observations on the order of a single gene to genome-wide studies and comparative genomics. The specifics differ in each case, but this full range of data is inherently complementary: drawn from living systems, it invites interrelation. However, such interrelation proves difficult because our methods for dealing with these data differ depending on their size and the scope of inquiry.

Ideally, scientists should record and share all useful findings. In reality however, results sometimes do not coincide with the standard ration suitable for journal publication. Some facts are simply too trivial to merit a whole paper, so isolated findings or negative results are often withheld from the published record. Conversely, some data sets are too large to include in article format. This latter problem is visible in a series of papers presenting whole-organism protein-protein interaction networks [1-4] or regulatory pathways [5-7]; these manuscripts present highlights and discussion, while the data sets themselves are stored in databases or on laboratory Web sites.

## A Changing Landscape: The Roles of Journals and Databases

Journals and databases are naturally positioned to handle different types and amounts of data. In addition, each offers a distinct and useful approach to the recording and transmission of scientific information.

Journal articles are optimized for human consumption. They are straightforward to create, intuitively organized and incorporate authoritative peer-review; however, they are not suited to handle very large or very small results and lack the rigorous organizational structure required for machine indexing and recall. Moreover, academic publishing is largely dominated by corporate publishers whose subscription barriers make third-party indexing of full-text material difficult or impossible. Nonetheless, journal articles are the established currency of scientific research: investigators must publish papers to advance their careers.

Databases are highly structured, efficient and machine-readable, but are not optimized for full-text discussion and require significant effort to establish and organize. They often lack true peer review – beyond a rudimentary oversight panel ensuring compliance with proprietary formats – and interrelating data between independent databases remains a challenge. Furthermore, contributing results even to flagship databases has yet to achieve similar status to publishing in a respected journal. Because no uniform citation system exists to track the database contributions of a given researcher (and little recognition is afforded to database deposit in general), very little professional incentive exists to populate, annotate or revise information stored in databases. As such, large teams of curators manually extract small-scale data from journal articles and deposit them in databases at great cost and effort (for example, the EBI sequence group has about 100 curators).

The once-sharp distinction between journals and databases is beginning to blur. We now search abstracts and access journal articles in a manner similar to database objects. Gone are the days of paging through an entire journal issue; now, articles are accessed through database-type portals such as PubMed, downloaded in PDF format and consumed piecemeal. The structure and format of the scholarly article should reflect this mode of access.

## A Future Vision

The optimal information architecture for biology would capture a broad range of data in digital format and facilitate database deposit alongside manuscript publication. It would index all full-text journal articles, associate keywords and identifiers with database records, and link textbooks, laboratory Web sites and high-level commentary. It would provide multiple levels of peer-review, community comment and annotation, and search results tailored to individual user profiles. This vast network of information would be interrelated, linked and accessed via a single seamless portal. Such a centralized system has been proposed before, and here we elaborate upon this vision [8].

From a technological standpoint the aforementioned functionality is entirely reasonable. Indeed, current technology allows for tremendous integration in the way we sift and access scientific data. (Journals in particular fail to exploit the full capability of the Internet, beyond including sporadic hyperlinks and employing the Web as a distribution medium for traditional text.) What is needed is a set of standards to actually accomplish this integration, which, in turn, requires the cooperation of disparate corporate and government entities – often entrenched and resistant to change.

Thus, as enticing as it is to imagine the vast functionality of a *de novo *information architecture designed to exploit modern digital technology, the reality is that journals and databases exist today in a form that does not allow instant adoption of such a highly-connected structure. Nonetheless, we can still aim to improve our current system while working toward an interconnected future. We have identified three key issues that we feel will lay the groundwork for tomorrow's expansive super-index while improving access to scientific information today.

## Toward a New Information Architecture – Three Key Issues

### 1. Expanded Publication Process

We must expand what it means to publish in the biosciences. Traditional journal articles alone are ill-suited to capture the fruits of modern research; and databases, Web sites, archived presentations and high-level commentaries are a valuable and real part of the scientific information landscape. Academic publication should reflect this.

To modernize academic publishing, we propose three main changes.

First, all scientists should be able to publish, share and access data on the Web. Funding agencies should include stable digital storage with research grants, and tie continued funding to the appropriate use of this storage. This ensures that every author is able to archive pre-prints, host supplementary and unpublished data, and make their findings widely available in digital format.

**Figure 1:**
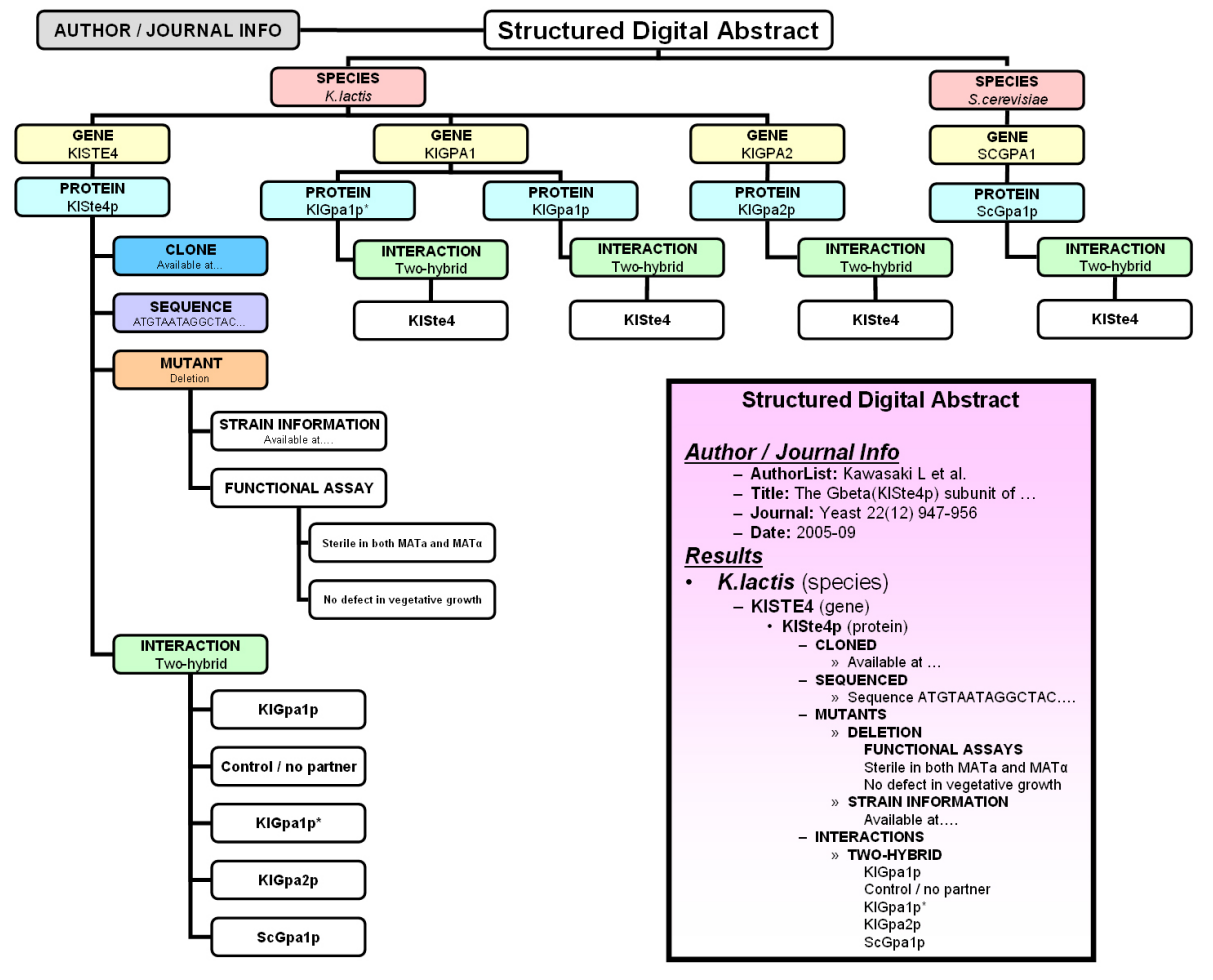
A schematic illustration of the proposed Structured Digital Abstract for a single genetics article [19]. This document – a machine-readable summary of pertinent findings arranged for simple database deposit – would be coded in XML and submitted alongside the manuscript for final publication. Inset; the same information presented in a hierarchical text-based format, similar to the final arrangement in the actual XML document.

Second, we propose that journals expand the publication process to yield a broader spectrum of output. Beyond the traditional text-based article, authors should produce two key products: a brief lay summary of their work (similar to those required by the journal *PLoS Medicine*), and a machine-readable XML summary of pertinent facts in the article which we term the Structured Digital Abstract (Figure 1). The former product assists public and non-specialist consumption of scientific research, and the latter would ease pressure on database curators and streamline the large-scale automatic deposition of author-vetted biological facts.

Third, findings should be contributed to an appropriate database when the paper is published. (This already occurs in several fields: in protein structure determination, for instance, deposition of data sets to the Protein Data Bank is a standard co-requisite for publication.) A Structured Digital Abstract will facilitate such deposit; individual journals could partner with related databases to decide on format and spearhead such practices. We might begin by using currently available information extraction software such as BioRAT [9] – a program that distills key facts from full-text documents in the biosciences – at the pre-print stage. Authors could build upon a BioRAT-style initial summary of their paper to create the final Structured Digital Abstract, submitted along with the manuscript for journal publication.

### 2. Central Indexing of Data

A key thrust of our future vision is the interconnection of disparate data sources: journal text is intelligently linked to database resources, third-party commentaries, archived talks and Web sites. To begin this process, we must establish a reliable way to identify database objects – similar to the Digital Object Identifier (DOI^®^) [10] already used for articles – and reference them consistently within journal text [11]. This conceptually simple idea will go a long way towards linking journal text with database information; for instance, a user browsing a given sequence feature in a database could instantly see and access all journal articles that reference that feature.

As a corollary, editorial boards should regulate a move towards a unified, standard naming convention for biology. The LSID proposal to unify database-specific ID-management issues into a single system and assign a unique identifier to all objects in the life sciences represents a promising advance towards such unified nomenclature.

Once consistently labeled, gene names and other biological identifiers in article text should be annotated for association with their database counterparts. This need not be overly tedious, as much of this process could be automated and implemented by journals during publication; a software system could identify most putative anchors and produce a checklist which authors would simply approve or alter [11]; the name lists (gazetteers) used by existing information extraction tools for biology will be useful here [9]. The end result would be journal text that comes pre-annotated with unique meta-identifiers, a suitable scaffold for the next generation of search and indexing.

The efficient interrelation of biological data sources has been the subject of much recent work. In particular, current applications of the semantic web to the biological world are promising: for instance, projects such as Atlas [12] (large-scale data integration infrastructure) and YeastHub [13] (using resource-description framework structures to warehouse tabular biological data) offer initial avenues for the handling of tomorrow's highly-annotated articles and data sets. Indeed, several prominent computer science researchers recently proposed the semantic web as the future of the Web in general [14].

Immediate gains will accompany Web search engines indexing the full text of scientific articles. Already underway to some degree with the Google Scholar service [15], this will rapidly expand search power beyond abstracts and keywords and dramatically improve public access to scientific information. It bears mention that such indexing is clearly dependent on some form of open access to scientific literature; initial efforts to index full-text must rely upon the cooperation of publishers, or free repositories such as PubMed Central and institutional archives.

Until widespread open access to published literature is a reality, local archiving on institutional Web space is a convenient stopgap to permit interim indexing of full-text documents. It is estimated that over 90% of academic journals allow some form of author archiving, but with widely differing rules [16]. Until these rules are standardized, publicized and well understood, authors will remain hesitant to archive. The Open Access movement is already pressing this issue, and we strongly support the wide adoption of Science Commons publication agreements [17], which clarify author rights relating to manuscript archiving.

### 3. Credit for Digital Contributions to Science

Scientific contribution should not be measured solely by journal publications. Database maintenance is already vital to modern research, and we should implement a consistent citation system to credit database contributions. Full-text publication will remain the cornerstone of the research process – after all, human-readable discussion will always be in high demand – but recognition should also be afforded to those who create, maintain and update the database records we depend upon daily. If database contribution is properly acknowledged, we will see more widespread attention devoted to maintaining these key resources in the future.

Moreover, the ability to quickly establish if an idea has previously been put forth – and to properly credit it, if so – is important to scientists. Full author identification and centrally searchable content will simplify this process, and facilitate attribution and acknowledgment.

Community annotation of published research is a key step towards harnessing the full power of the Internet in scientific communication. The new journal *PLoS One *[18] offers community-driven peer-review, permitting online discussion and rating of work by a wide spectrum of interested parties. With a tangible model for open review in place, it will be interesting to observe the success of this approach, and whether other publishers follow suit.

Finally, overall progress toward our future vision will likely change how we view authoring and editing in science. Specifically, curating biological databases is of increasing importance. This complex task demands scientific expertise paired with writing, editing, programming and database administration skills. We believe data management techniques will one day be taught to undergraduate-level scientists, as students and researchers of all levels learn to oversee and tend their corner of the digital data landscape.

## Conclusion

The scholarly publishing industry remains heavily invested in a dwindling share of today's scientific information landscape: the traditional journal. Change is in order. This does not mean we should rush headlong into building a massive digital index, for technologies must mature and stabilize to support such a system. Rather, it is time to recognize the centrality of biological databases to modern research, and work to better integrate them with the vast corpus of knowledge contained in journals, news sources, commentaries and talks. To achieve this, we must consider how best to apply current technology to the task of capturing and communicating scientific information. By overhauling the publication process today, we not only improve our current handling of scientific information; we invest in a connected future, an information architecture capable of linking and interrelating knowledge like never before.

## Authors' contributions

MS and MG conceived of the commentary and collaborated in writing the article.
